# Activation of the Transducers of Unfolded Protein Response in Plants

**DOI:** 10.3389/fpls.2018.00214

**Published:** 2018-02-20

**Authors:** Ganesh M. Nawkar, Eun Seon Lee, Rahul M. Shelake, Joung Hun Park, Seoung Woo Ryu, Chang Ho Kang, Sang Yeol Lee

**Affiliations:** Division of Applied Life Sciences (BK21 Plus) and Plant Molecular Biology and Biotechnology Research Center (PMBBRC), Gyeongsang National University, Jinju, South Korea

**Keywords:** endoplasmic reticulum, abiotic/biotic stress, UPR activation, bZIP28, bZIP60, IRE1, NAC-TFs

## Abstract

Maintenance of homeostasis of the endoplasmic reticulum (ER) ensures the balance between loading of nascent proteins and their secretion. Certain developmental conditions or environmental stressors affect protein folding causing ER stress. The resultant ER stress is mitigated by upregulating a set of stress-responsive genes in the nucleus modulating the mechanism of the unfolded protein response (UPR). In plants, the UPR is mediated by two major pathways; by the proteolytic processing of bZIP17/28 and by the IRE1-mediated splicing of *bZIP60* mRNA. Recent studies have shown the involvement of plant-specific NAC transcription factors in UPR regulation. The molecular mechanisms activating plant-UPR transducers are only recently being unveiled. This review focuses on important structural features involved in the activation of the UPR transducers like bZIP17/28/60, IRE1, BAG7, and NAC017/062/089/103. Also, we discuss the activation of the UPR pathways, including BAG7-bZIP28 and IRE1-bZIP60, in detail, together with the NAC-TFs, which adds a new paradigm to the plant UPR.

## Introduction

**FIGURE 1:**
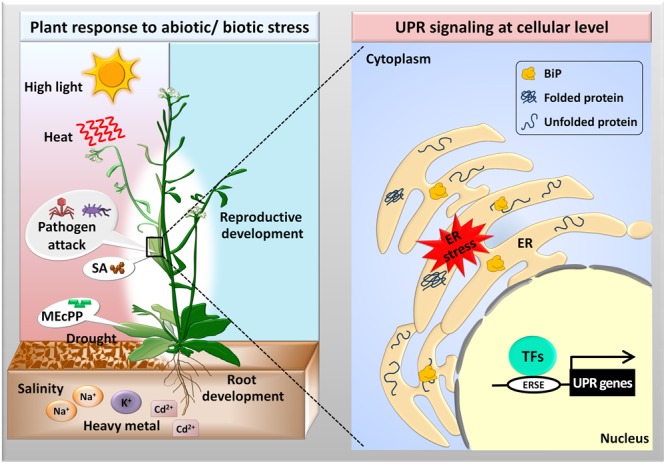
Activation of the unfolded protein response (UPR) in Arabidopsis in response to external stresses. Abiotic (high light intensity, temperature, salt, drought, and heavy metals) and biotic (pathogenic attack) stresses disturb protein folding in the ER lumen causing the activation of transcription factors (TFs), which bind to the ER stress response elements (ERSEs) to upregulate the UPR genes. The UPR plays an important role during vegetative root growth and reproductive development in Arabidopsis. Moreover, plant signaling compounds such as 2-C-methyl-D-erythritol-2,4-cyclopyrophosphate (MEcPP) and salicylic acid (SA) activate the UPR pathways via unknown mechanisms.

In eukaryotes, the endoplasmic reticulum (ER) acts as a factory site for proper folding and maturation of secretory and membrane proteins, which comprise about one-third of the total proteome (Wallin and von Heijne, 1998). These proteins undergo post-translational modification, such as N-linked glycosylation and disulfide bond formation, in the ER lumen (Braakman and Bulleid, 2011). ER-factory is well-equipped with a protein folding machinery containing molecular chaperones, including the luminal binding protein (BiP), calnexin (CNX), and calreticulins (CRT), and folding enzymes like the protein disulfide isomerase (PDI). Molecular chaperones prevent the aggregation of denatured proteins and assist in their proper folding, while PDI catalyzes the formation of correct disulfide bridges between the cysteine residues in proteins to maintain ER-homeostasis (Vitale and Denecke, 1999; Park and Seo, 2015). In plants, abiotic stressors, including high temperature, salt, osmotic stress, drought, heavy metals, and high light intensity, or biotic agents, such as viruses, disturb protein folding (**Figure 1**) (Liu et al., 2007b; Gao et al., 2008; Valente et al., 2009; Liu and Howell, 2010b; Deng et al., 2011; Faria et al., 2011; Mendes et al., 2013; Korner et al., 2015; Verchot, 2016; Xi et al., 2016; Nawkar et al., 2017). As a consequence, the accumulation of unfolded or misfolded proteins exceeds the ER-protein folding capacity leading to ER stress conditions (Liu and Howell, 2010b; Deng et al., 2011). During the last decade, there have been lots of development in the field of plant-ER stress signaling and unfolded protein response (UPR) modulated by a set of stress-responsive genes in the nucleus (Liu and Howell, 2016; Wan and Jiang, 2016; Angelos et al., 2017). Primarily, to overcome ER stress, plants activate the conserved UPR mechanism (**Figure 1**). The UPR plays an important role in restoring the protein folding capacity of the ER membrane by increasing the levels of molecular chaperones, and reducing the protein load by enhancing ER-associated protein degradation (ERAD) (Liu and Howell, 2016). Moreover, the UPR supports vegetative root growth and plays a vital role during the development of the male reproductive parts of a plant, not only under normal but also under stress conditions (Deng et al., 2013; Bao and Howell, 2017). In particular, the UPR maintains plant fertility by regulating pollen development during stressful conditions (Fragkostefanakis et al., 2016; Zhang et al., 2017). Recently, it has been suggested that the stress-responsive, plastid originated retrograde signal, 2-C-methyl-D-erythritol-2,4-cyclopyrophosphate (MEcPP), and the plant defense hormone, salicylic acid (SA), induces UPR in plants, although the underlining mechanism is not yet completely known (Nagashima et al., 2014; Walley et al., 2015). Generally, under laboratory conditions, the UPR has been studied using several chemicals interfering with the post-translational protein modifications; examples include the inhibition of N-glycosylation by tunicamycin (Tm), hindrance to the formation of disulfide bonds by dithiothreitol (DTT), and interference in the formation of native protein structures by the proline homolog azetidine-2-carboxylic acid (AZC) (Howell, 2013).

In plants, the processes of regulated intramembrane proteolysis (RIP) of basic leucine zipper (bZIP) transcription factors (TFs) like *bZIP17/28*, and the *inositol-requiring enzyme 1 (IRE1)*-mediated unconventional splicing of *bZIP60* act as the primary pathways of the UPR (Iwata and Koizumi, 2012). The activation of the membrane-tethered TFs (MTFs) by the RIP and the activation of the IRE1-dependent pathway are conserved in plants, mammals, and fungi (Chakraborty et al., 2016). Recently, the plant B-cell lymphoma2 (Bcl-2)-associated athanogene 7 (BAG7) protein, belonging to an evolutionarily conserved family of co-chaperones, has been shown to be involved in the regulation of the heat-induced UPR pathway (Li et al., 2017). The protein kinase RNA-like ER kinase (PERK)-mediated translational inhibition was characterized in mammals (Ruberti and Brandizzi, 2014). Also, the plant-specific NAC [acronym derived from No apical meristem (NAM), Arabidopsis transcription activation factor (ATAF), and Cup-shaped cotyledon (CUC)]-TFs are shown to be involved in ER stress response (Sun et al., 2013; Yang et al., 2014b; Chi et al., 2017). These NAC-TFs are equivalent to the secondary UPR transducers in plants. The knowledge about the activation mechanisms of the UPR in model plants such as Arabidopsis is a prerequisite for developing stress-tolerance in the agriculturally important crops. In this review, we have summarized the structural features of individual UPR-sensors and focused on the mechanistic insights into the activation of the conserved arms of the UPR, such as the bZIP28 and IRE1-bZIP60 pathways, as well as on the plant-specific UPR transducers including NAC-TFs.

## The Mechanism of Activation of bZIP28

**FIGURE 2:**
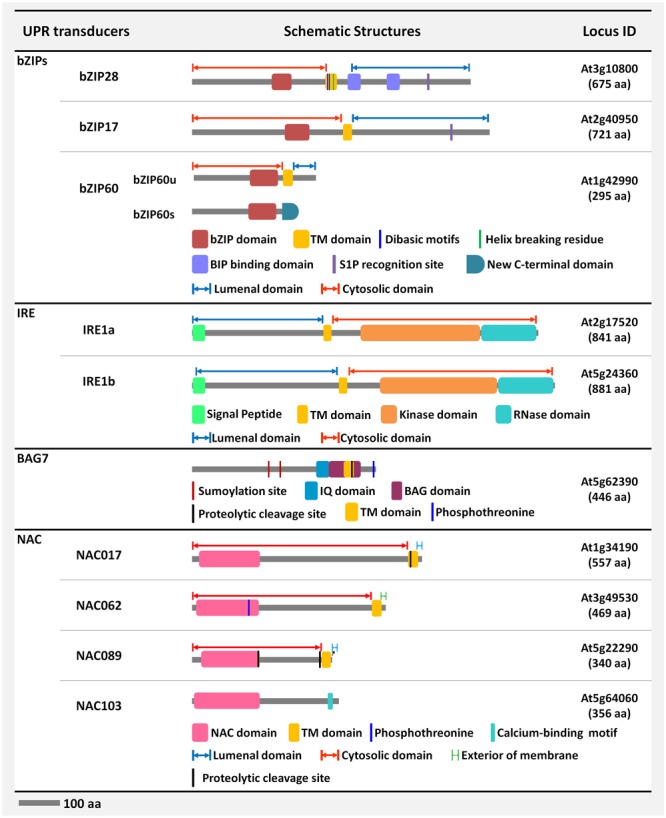
Schematic representation of the structural features of the signal transducers of the unfolded protein response (UPR) in Arabidopsis. The molecular structures of the Arabidopsis signal transducing elements in the UPR pathway are schematically represented. The structural features of the bZIP/NAC-TFs, coregulators-BAG7, and dual-functioning enzymes-IRE1 are briefly depicted.

**FIGURE 3:**
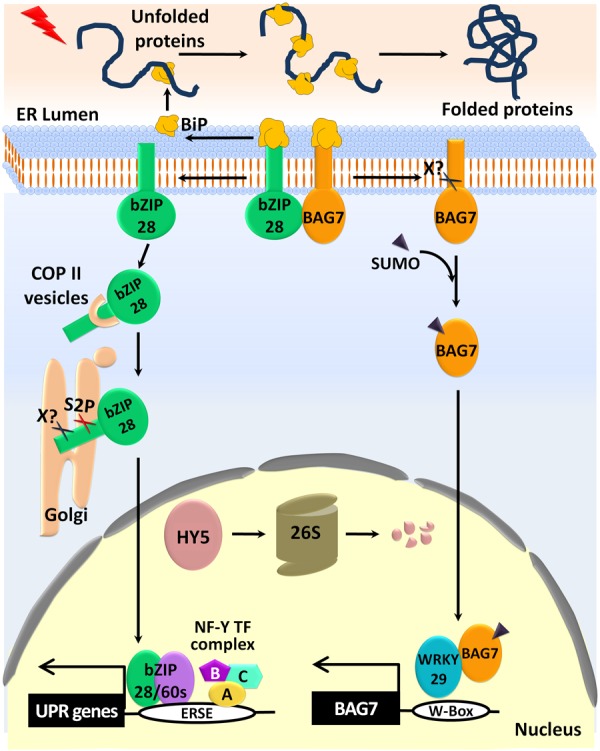
ER stress induced activation of bZIP28 and BAG7 in plant UPR signaling. Under normal conditions, the bZIP28 and BAG7 proteins are anchored to the ER membrane by interactions with the Binding protein (BiP). However, in response to ER stress, BiP assists the proper folding of the unfolded proteins. Then the released bZIP28 traffics to the Golgi through the Coat Protein II (COPII) vesicles. After the proteolytic cleaving of the bZIP28 by an unknown protease (X?) and by the site-2 protease (S2P), the truncated active form of bZIP28 translocates into the nucleus. The nuclear bZIP28 outcompetes HY5 for binding to the ERSE element and forms a transcriptional complex with bZIP60 and NF-Y-TFs to activate the UPR gene expression. The 26S-proteasomal degradation system eventually degrades the released HY5. Additionally, BAG7 is also released from the ER membrane by an unknown protease (X?). Subsequently, BAG7 is sumoylated and enters the nucleus. The nuclear BAG7 interacts with WRKY29 and regulates the expression of BAG7 and other chaperone proteins to mitigate ER stress.

To mitigate ER stress in plants, the RIP-mediated activation of the bZIP28 is the first-hand response. The molecular structure of bZIP28, a type II membrane protein, shows that it contains a single transmembrane domain (TMD), a cytoplasmic DNA-binding bZIP domain at its N-terminus, and a luminal domain at its C-terminus (**Figure 2**) (Liu et al., 2007a). During the past decade, accumulating evidence suggested the step-wise activation of a bZIP28 in response to ER stress (**Figure 3**). Under an unstressed condition, the master regulator BiP binds to an intrinsically disordered region of the luminal domain of bZIP28, while it dissociates from the bZIP28 in response to ER stress (Srivastava et al., 2013). Once released from BiP, bZIP28 interacts with Sar1a through its dibasic motifs (KK 311, 320) present in its cytosolic domain, to initiate vesicle formation with coat protein complex II (COPII) (Srivastava et al., 2012). Under ER stress conditions, the transcriptionally induced COPII components, Sar1a/Sec23a, promote the trafficking of bZIP28 from the ER to the Golgi complex (Song et al., 2015; Zeng et al., 2015). Site-1 protease (S1P) is not involved in the processing of bZIP28 in the Golgi body, despite the presence of the consensus S1P recognition motif (RRIL) at its luminal domain. Rather, as-yet unidentified protease(s) first cleaves the bZIP28 at its TMD making it available as a substrate for the site-2 protease (S2P) (Iwata et al., 2017). S2P recognizes the putative helix-breaking residue (G329) in the TMD of bZIP28 for proteolytic processing, and the active cytosolic bZIP28 translocates to the nucleus (Srivastava et al., 2012). The nuclear-localized bZIP28 forms a transcriptional complex with the nuclear factor-Y (NF-Y) TFs, and binds specifically to the ER stress response element (ERSE) that is located in the promoter regions of the UPR genes (Liu and Howell, 2010a).

In addition to the RIP-induced activation of the bZIP28 pathway of plant UPR, different layers of regulation cascade in the bZIP28-mediated UPR activation have been identified. Firstly, the retention of bZIP28 by the master regulator BiP under unstressed conditions is assisted by the co-chaperone function of the BAG7 protein located in the ER membrane. The BAG7 protein facilitates the direct interaction of bZIP28 with BiP under unstressed conditions. However, similar to bZIP28, ER stress also triggers a proteolytic release of the BAG7 protein, which is then sumoylated and translocated to the nucleus where it interacts with TF WRKY29 that regulates the stress-responsive genes (Williams et al., 2010; Li et al., 2017). Secondly, the activity of bZIP28 as a TF for the UPR is inhibited by another bZIP-TF, elongated hypocotyl 5 (HY5) under unstressed conditions, since they compete for binding to the G-box element (CACGTG) present at the ERSE motifs in the promoters of the UPR genes. Under ER stress conditions, the negative regulator of UPR, HY5, undergoes proteasomal degradation. After that, HY5 is out-competed by the nuclear-localized bZIP28 for binding to the ERSE motif, and activates the UPR (Nawkar et al., 2017). Moreover, the interaction of bZIP28/60 with the core components of the COMPASS-like complex mediates chromatin remodeling of the active promoters of the UPR genes through the sequence-specific histone H3K4 trimethylation (H3K4me3). This chromatin modification is critically essential for the up-regulation of the UPR genes (Song et al., 2015).

## Activation of the IRE1-bZIP60 Pathway

In many eukaryotes, activation of the IRE1-mediated unconventional splicing of mRNA is the most conserved arm of the UPR (Chen and Brandizzi, 2013; Ruberti et al., 2015). In Arabidopsis, there are two isoforms of *IRE1*-*IRE1a* and *IRE1b* (Koizumi et al., 2001). Both the isoforms, IRE1a and IRE1b, are classified as type I single-pass transmembrane proteins. They consist of multi-functional domains such as an N-terminal signal-peptide, an ER-stress sensing domain facing the ER lumen, a protein kinase domain, and a C-terminal ribonuclease domain facing the cytosol (**Figure 2**). In response to biotic and abiotic stresses, *IRE1a* and *IRE1b* specifically activate the unconventional splicing of the *bZIP60* mRNA (Deng et al., 2011; Moreno et al., 2012). The plant IRE1a and IRE1b can form homo/heterodimers to enable the activation of the IRE1-dependent UPR signaling pathway, similar to the activation of the IRE pathway in yeast and mammals (Credle et al., 2005; Zhou et al., 2006; Zhang et al., 2015). The yeast IRE1p activation is a stepwise mechanism that is fine-tuned by the binding of BiP to the luminal domain of IRE1p (Pincus et al., 2010). Importantly, the luminal IRE1a/1b sensor domains of plants functionally complement the yeast IRE1p sensor domain and rescue the ER stress-sensitive phenotype of the *Δire1* yeast mutant (Koizumi et al., 2001). Under ER stress conditions, the luminal domain of the yeast IRE1p forms a composite groove structure by dimerization, where unfolded proteins bind and trigger IRE1p oligomerization and clustering (Korennykh et al., 2009; Gardner and Walter, 2011). Although the exact mechanism of IRE1 activation in plants has not been elucidated yet, the Arabidopsis IRE1b, but not IRE1a, showed the oligomerization structure and clustering when expressed heterologously in the yeast cells. It indicates that the first step of IRE1 activation is conserved not only in yeast, and mammals but also in Arabidopsis (Zhou et al., 2006; Zhang et al., 2016). Proper clustering and activation of Arabidopsis IRE1 requires the function of the ER-shaping GTPase *Root Hair Defective 3* (*RHD3*), of which the underlining mechanism of action is unknown (Lai et al., 2014). Once activated, the yeast IRE1p aligns its cytosolic kinase domains in such a way that they trans-autophosphorylate each other resulting in conformational changes. The altered confirmation leads to the activation of the RNase domain of the yeast IRE1p enzyme (Shamu and Walter, 1996; Lee et al., 2008; Korennykh et al., 2011). Based on the *in vitro* kinase activity of the plant IRE1 and the presence of a higher degree of conserved cytosolic kinase and RNase domains among the eukaryotes, it is believed that the plant IRE1 also gets activated by trans-autophosphorylation similar to the yeast IRE1p (Koizumi et al., 2001; Zhang et al., 2016).

**FIGURE 4:**
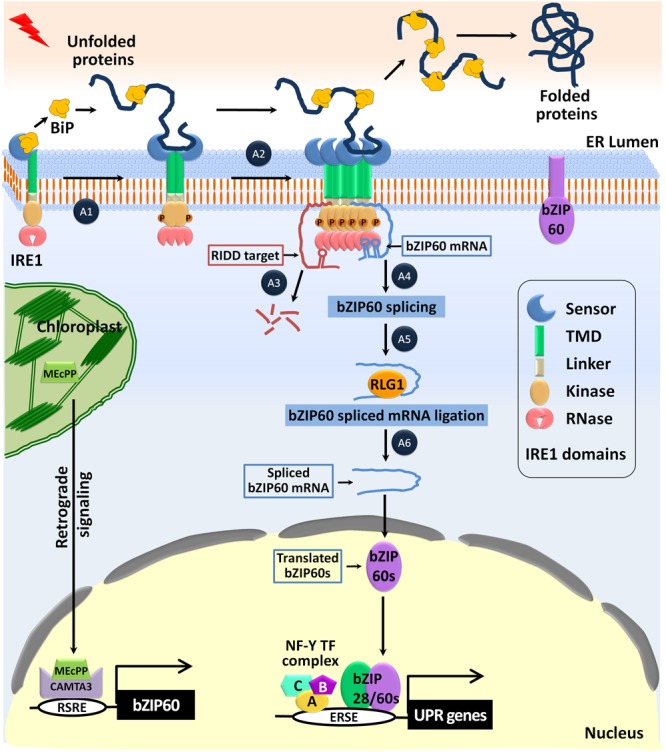
Activation of the IRE1-bZIP60 mediated UPR pathway against ER stress. Under normal conditions, the sensor domain of IRE1 binds to the ER-luminal Binding protein (BiP) and the full-length bZIP60 is anchored in the ER membrane. In response to ER-stress, 2-C-methyl-D-erythritol-2,4-cyclopyrophosphate (MEcPP) is accumulated in the chloroplast and activates the *Calmodulin-Binding Transcription Activator 3 (CAMTA3)*-TF, which binds to the rapid stress response element (RSRE) and up-regulates *bZIP60* expression. A1–A6 indicates the step-wise activation process of IRE1. (A1) In response to ER stress, BiP dissociates from IRE1 to assist the proper folding of the accumulated unfolded proteins. The released IRE1 is dimerized to align its cytosolic kinase domains in such a way that they trans-autophosphorylate each other to activate the RNase function. (A2) The luminal domain of IRE1 binds to the hydrophobic domain of the unfolded proteins and triggers oligomerization and clustering. (A3) The mRNAs encoding the secretory pathway proteins are promiscuously cleaved by the proteins of the regulated IRE1-dependent decay (RIDD) pathway. (A4) The basic linker region of IRE1 mediates specific recruitment and docking of the unspliced *bZIP60* mRNA to IRE1 clusters where it is cleaved explicitly at the two conserved sites (CXG| XXG) present in the kissing stem–loop structure. (A5) The tRNA ligase RLG1 catalyzes the ligation of the resulting fragments of the *bZIP60* mRNA. (A6) Translation of the spliced *bZIP60* mRNA results in the production of the active bZIP60 protein (bZIP60s). It translocates to the nucleus forming the bZIP28-NF-Y-TFs complex. These TF-complexes bind to the ERSE element of the UPR genes and positively regulate the UPR-related downstream gene expression.

The yeast IRE1p clusters mediates the selective recruitment of a bipartite element in the 3′-untranslated region of the *HAC1* (*homologous to ATF/CREB 1*) mRNA. Then the positive charge of a cytosolic linker in the yeast IRE1p mediates mRNA docking, which is an essential step for the unconventional splicing of the *HAC1* mRNA (Aragon et al., 2009). In Arabidopsis, the selective targeting and docking of the unspliced *bZIP60* mRNA toward the IRE1 clusters have not been reported. However, in the case of plants, it is postulated that the bZIP60 protein with a hydrophobic membrane-anchoring domain mediates the pre-recruitment of mRNA to the ER membrane. Furthermore, the conserved basic linker motif of the IRE1 homologs in plants serves as an mRNA docking site, which needs more experimental validations (van Anken et al., 2014). Similar to that of the yeast IRE1p, the activated RNase function of the Arabidopsis IRE1a/b mediates the unconventional splicing of the *bZIP60* mRNA at two conserved sites (CXG| XXG) present in the kissing stem–loop structure. It results in the release of a single intron, and the spliced-fragments of the *bZIP60* mRNA are ligated by the tRNA ligase RLG1 in the cytoplasm (**Figure 4**) (Sidrauski et al., 1996; Sidrauski and Walter, 1997; Deng et al., 2011; Nagashima et al., 2011, 2016). Upon translation of the spliced mRNA, the active form of bZIP60 without TMD (i.e., bZIP60s) translocates into the nucleus and binds to the promoter region of the UPR genes to up-regulate the UPR genes (Walter and Ron, 2011; Iwata and Koizumi, 2012). Apart from the unspliced *bZIP60* mRNA, the Arabidopsis IRE1 also targets the degradation of the mRNAs encoding the proteins functioning in a secretory pathway designated as the Regulated IRE1-Dependent Decay (RIDD) of mRNAs pathway (Mishiba et al., 2013). To mediate the degradation of the mRNAs, the mammalian IREα recognizes the consensus mRNA sequence (CUGCAG) accompanied by a stem–loop structure (Oikawa et al., 2010). It will be interesting to identify such a structural specificity for the Arabidopsis IRE1-RIDD targets.

In the beginning, Arabidopsis *bZIP60* was characterized as a Tm-induced bZIP-TF, which regulates the UPR. Moreover, *bZIP60* induces its transcription through an ERSE-like sequence present in the promoter region (Iwata and Koizumi, 2005). Recently, MEcPP, a metabolite functioning in stress-specific retrograde signaling, has also been shown to be involved in the regulation of *bZIP60* at the transcript level (Benn et al., 2016). MEcPP is a stress-induced isoprenoid intermediate produced by the methylerythritol phosphate (MEP) pathway (Xiao et al., 2012). MEcPP positively regulates the general stress response (GSR) by activating the *Calmodulin-Binding Transcription Activator 3 (CAMTA3)*-TF, which binds to a rapid stress response element (RSRE). MEcPP-CAMTA3 induces the expression of *IRE1* and *bZIP60* through RSRE, following which the complex initiates the GSR, an essential process for restoration of ER-homeostasis (**Figure 4**) (Walley et al., 2015; Benn et al., 2016).

## Activation of the NAC-TFs Pathway

The plant-specific NAC proteins belong to one of the largest gene families of TFs (Jensen et al., 2010). In the Arabidopsis genome, about 117 NAC genes have been identified, out of which 13 are predicted to be type II membrane-associated proteins (Seo et al., 2008; Puranik et al., 2012). These NAC-TFs are involved in various developmental pathways and phytohormonal interplay, and are upregulated at the transcription level by different abiotic stresses (He et al., 2005; Jiang and Deyholos, 2006; Kim et al., 2007; Tran et al., 2007). The NAC-MTFs are proteolytically processed in response to different stress stimuli such as cold, drought, high salinity, osmotic stress, or hormonal stimuli, including abscisic acid (ABA), gibberellic acid (GA), and cytokinin. Regulated activation of the MTFs is essential for rapid stress-response and plant development (Kim et al., 2008; Yoon et al., 2008; Seo et al., 2010b).

**FIGURE 5:**
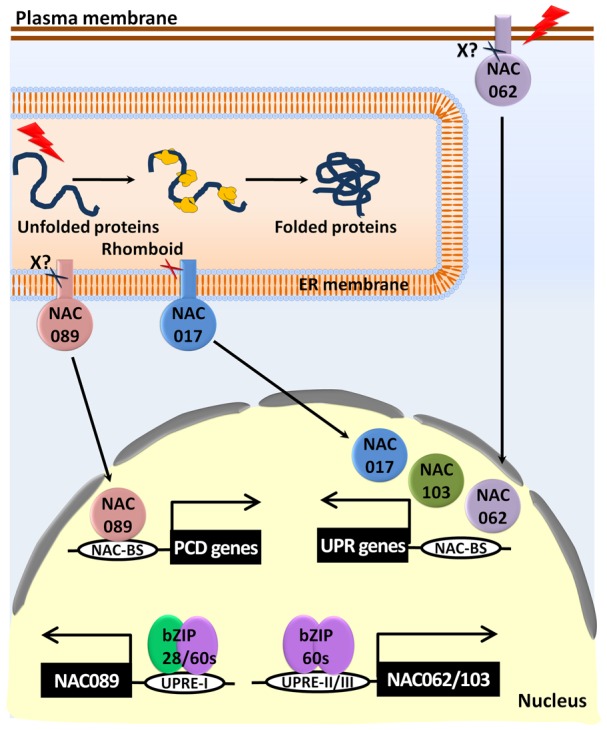
Activation of the NAC-TFs mediated UPR signaling pathways in response to ER stress. In response to ER stress, bZIP60s binds to the UPR element-II/III (UPRE-II/III) to transcriptionally upregulate the expression of NAC062 and NAC103. An unknown protease activates the plasma membrane-localized NAC062. A rhomboid protease is involved in the activation of the ER membrane-localized NAC017. The nuclear localized-NAC103 along with the activated NAC017/062 binds to the NAC-binding sites and activates the UPR gene expressions. Under chronic ER stress conditions, bZIP28/60s binds to the UPRE-I element of the *NAC089* promoter and activates its gene expression. Moreover, NAC089 is processed by an unknown protease and translocates to the nucleus to upregulate the genes involved in programmed cell death (PCD).

Difference in the stresses affecting protein folding leads to variations in the ER stress. The induction of stress-specific NAC-TFs in plants is needed to rapidly up-regulate the UPR downstream genes to withstand these stress conditions. For example, the NAC-TFs *NAC017, NAC062, NAC089*, and *NAC103* are activated in response to the canonical ER stress-inducing agents such as Tm and DTT (**Figure 5**) (Sun et al., 2013; Yang et al., 2014b; Chi et al., 2017). The mitochondrial retrograde signaling component NAC017, which has a predicted TMD, is localized in the connections and junctions of the ER and F-actin. It acts as a high-level transcriptional regulator of the H_2_O_2_-mediated primary stress response in plants. The presence of a consensus rhomboid protease cleavage site (LSIVGA) just before the TMD of the NAC017-TF regulates its activation under stress conditions (Ng et al., 2013). Furthermore, the active NAC017 without TMD imparts ER stress tolerance (Chi et al., 2017). The expression of *NAC062* and *NAC103* is induced by bZIP60 through direct binding to UPRE III (TCATCG) present in their *cis*-elements (Sun et al., 2013; Yang et al., 2014a). NAC062 is localized to the plasma membrane and activated under cold/pathogen stresses to regulate the expression of the *PR1* genes under stress conditions (Seo et al., 2010a). Although the exact mechanism for the proteolytic activation of NAC062 is poorly understood, it has been suggested that changes in the lipid composition of the plasma membrane due to ER stress or cold stress triggers the proteolytic cleavage of NAC062 by unknown zinc-dependent proteases (Seo et al., 2010b). The overexpression of NAC062 lacking the TMD and the nuclear-localized NAC103 involved in the up-regulation of the UPR genes positively mediate ER stress tolerance (Sun et al., 2013; Yang et al., 2014a). Interestingly, the overexpression of the other ER-localized protein NAC089 without TMD, results in programmed cell death (PCD), while the knock-down of its coding gene imparts ER stress tolerance (Yang et al., 2014b). Both bZIP28 and bZIP60 are involved in the upregulation of the *NAC089* transcript under ER stress condition, but the protease enzyme involved in the activation of NAC089 is still unknown. Two cleavage sites in the NAC089 sequence at the amino acid position 163 (VVCRVRR| NK), a particular Arg/Lys-specific site, and a second site at position 297 (RPSQKKK| GK) have been predicted. An active NAC089 regulates the genes involved in PCD such as *NAC094, metacaspase 5 (MC5)*, and *BAG6* (Yang et al., 2014b). Moreover, the redox-dependent NAC089 functions as a negative regulator of the *stromal ascorbate peroxidase (sAPX)* gene expression, which is involved in the chloroplast antioxidant defense system (Klein et al., 2012; Yang et al., 2014b).

## Concluding Remarks

In Arabidopsis, all of the UPR transducers possess a TMD and are localized in the ER membrane, except NAC103 and NAC062. NAC103 is a constitutively nuclear-localized protein lacking a TMD, while NAC062 is a plasma membrane-localized protein. The lengths of the luminal domains vary among the sensor/transducer proteins, which results in the complexity in understanding the mechanism of UPR activation. For example, the interaction of the master regulator BiP with a luminal domain of bZIP28, BAG7, and IRE1 acts as a switch in the bZIP28-BAG7 and IRE1-bZIP60 pathways, respectively. Contrarily, the NAC-TFs have a shorter luminal domain and their interaction with BiP is not yet reported. Therefore, information about the key regulators in the NAC-TF-mediated pathway is still missing. For a rapid response, the MTFs are regulated at two levels; first at the post-translation level, second at the transcript level. The MTFs are located in the membrane and activated upon the onset of stress. The regulated activation of the MTFs depends on various proteases that are mostly unknown. Identification of the proteases processing specific MTFs under certain physiological conditions will help to understand the cross-talk between the activation pathways of the UPR and signaling by hormones including auxin, brassinosteroids, and SA. The advancement in the understanding of the relationship between proteases and MTFs will be helpful in plant biotechnology to improve the stress-tolerance of crop varieties and in molecular pharming.

## Author Contributions

GN, CHK, and SYL conceived the idea and designed the outlines of the article. GN, ESL, RS, JHP, and SWR wrote the article. GN, RS, CHK, and SYL revised the article.

## Conflict of Interest Statement

The authors declare that the research was conducted in the absence of any commercial or financial relationships that could be construed as a potential conflict of interest.
